# Encapsulation of Cannabidiol in Chitosan-Stabilized Argan Oil Nanoemulsion as a Potential Dermal Drug Delivery System for Psoriasis Treatment

**DOI:** 10.3390/pharmaceutics18030286

**Published:** 2026-02-26

**Authors:** Yousra Mdarhri, Vinicius de-Monte-Vidal, Camila de-Almeida-Perez-Pimenta, Selene Cuello-Rodríguez, Ahmed Touhami, Fakhita Touhami, Mohamed Chabbi, Victoria Díaz-Tomé

**Affiliations:** 1 Department of Chemistry, Faculty of Science and Technology of Tangier, Abdelmalek Essaâdi University, Tetouan 93000, Morocco; yousra.mdarhri@gmail.com (Y.M.); mchabbi@uae.ac.ma (M.C.); 2 Department of Pharmacology, Pharmacy and Pharmaceutical Technology, University of Santiago de Compostela, 15705 Santiago de Compostela, Spain; vinicius.demonte@rai.usc.es (V.d.-M.-V.); mila.perez.pimenta@gmail.com (C.d.-A.-P.-P.); selene.cuello@rai.usc.es (S.C.-R.); 3 Institute of Materials iMATUS, University of Santiago de Compostela, 15706 Santiago de Compostela, Spain; 4 Paraquasil Group, University Clinical Hospital, Health Research Institute of Santiago de Compostela, 15706 Santiago de Compostela, Spain; 5 Department of Pharmaceutical Sciences, Center for Pharmaceutical and Cosmetic Development, Federal University of Pernambuco, Recife 50740-521, PE, Brazil; 6 Department of Physics & Astronomy, College of Sciences, University of Texas Rio Grande Valley, Edinburg, TX 78539, USA; ahmed.touhami@utrgv.edu; 7 Information and Communication Systems Department, National School of Applied Sciences of Tangier, Abdelmalek Essaâdi University, Tetouan 93000, Morocco; f.touhami@uae.ac.ma

**Keywords:** cannabidiol, nanoemulsion, chitosan, argan oil, topical drug delivery, psoriasis treatment

## Abstract

**Background**: Cannabidiol (CBD), a non-psychoactive compound derived from *Cannabis sativa*, exhibits therapeutic potential for various conditions, including inflammation, pain, and skin disorders, making it a promising candidate for the topical treatment of psoriasis. However, its poor water solubility and instability limit its therapeutic efficacy. This study focuses on the development and characterization of CBD-loaded nanoemulsions using argan oil as the lipid phase, with and without a chitosan coating, which serves as a stabilizing and functional biopolymer. **Methods**: Nanoemulsions (NE) and chitosan-stabilized nanoemulsions (CS-NE), both without CBD (serving as controls), and CBD-loaded variants (CBD-NE and CBD-CS-NE) were prepared and characterized for their physicochemical properties, including pH, droplet size, polydispersity index (PDI), zeta potential (ζ-potential), and viscosity at various shear rates and temperatures. Stability was assessed over time, and drug release behavior was investigated through *in vitro* diffusion and *ex vivo* skin permeation studies, followed by kinetic modeling. Safety was evaluated through *in vitro* cytotoxicity assays using HaCaT keratinocyte cells, as well as *in vivo* toxicity studies using *Caenorhabditis elegans* (*C. elegans*). **Results**: The chitosan-coated formulations exhibited enhanced physical stability, nanoscale droplet size, a positive surface charge, and increased viscosity. Release studies demonstrated that CBD-CS-NE enabled controlled and sustained drug release, with a strong correlation to the Higuchi model, indicating diffusion-controlled permeation. Cytotoxicity assays indicated that CBD-CS-NE was non-toxic to cultured cells, while *in vivo* testing with *C. elegans* revealed sensitivity to chitosan-coated systems. **Conclusions**: These findings highlight the potential of CBD-loaded argan oil nanoemulsions, particularly those stabilized with chitosan, as potential topical delivery systems for managing psoriasis.

## 1. Introduction

Dermatological disorders encompass a wide range of conditions affecting the skin, hair, and nails, often significantly impacting an individual’s physical and psychological well-being. Among these, Inflammatory Skin Diseases (ISDs) are particularly challenging, as they involve complex immune responses and persistent symptoms such as redness, itching, and discomfort. Psoriasis, a prevalent chronic inflammatory skin disease, exemplifies such conditions [1]. Characterized by the rapid proliferation of keratinocytes and immune-mediated inflammation, psoriasis manifests as well-defined, scaly, erythematous plaques, typically appearing on the scalp, elbows, knees, and lower back. Beyond its physical symptoms, psoriasis is associated with systemic complications, including psoriatic arthritis and an increased risk of cardiovascular and metabolic disorders [2]. Despite advancements in treatment options, managing this chronic inflammatory condition remains difficult, emphasizing the need for innovative therapeutic strategies to improve patient outcomes and quality of life.

In recent years, Cannabis-Based Medicines (CBMs) have garnered significant attention for their potential therapeutic applications across a wide range of medical conditions. Derived from the *Cannabis sativa* plant [3], these pharmaceutical products utilize cannabinoids as their active ingredients, with CBD emerging as a particularly promising compound. CBD, a non-psychoactive cannabinoid, is renowned for its pharmacological benefits, including anti-inflammatory, immunomodulatory, and skin-soothing properties, positioning it as a viable candidate for addressing psoriasis [4,5]. Emerging research highlights CBD’s potential in managing this chronic skin condition through multiple mechanisms, including reducing inflammation and oxidative stress, alleviating itching, inhibiting abnormal keratinocyte proliferation, and enhancing skin hydration [2]. Together, these attributes underscore the therapeutic promise of CBD in improving both the symptoms and quality of life for individuals with psoriasis. However, the effective delivery of CBD is hindered by its poor water solubility, low bioavailability, and vulnerability to oxidative degradation, which collectively limit its therapeutic effectiveness [6].

Nanoemulsions have emerged as advanced drug delivery systems capable of overcoming these limitations. These colloidal dispersions, characterized by their small droplet size and high surface area, provide a means to encapsulate hydrophobic drugs, enhancing their solubility, stability, bioavailability, and dermal penetration of bioactive compounds [7]. Argan oil, valued for its emollient and skin-healing properties, serves as an ideal oil phase in such formulations [8], further complementing the therapeutic benefits of CBD.

In this study, chitosan, a biodegradable and biocompatible polymer derived from natural sources, is employed as a stabilizing agent to address formulation stability challenges [9]. Chitosan not only enhances the physical stability of nanoemulsions and facilitates sustained release but can also improve permeation through biological membranes. Furthermore, it possesses intrinsic antibacterial and wound-healing properties, making it an ideal component for dermal drug delivery systems, particularly for psoriasis treatment [10].

This study aims to develop and comprehensively evaluate a chitosan-stabilized argan oil nanoemulsion for the dermal delivery of cannabidiol (CBD) as a potential therapy for psoriasis. The central hypothesis is that the chitosan-stabilized nanoemulsion will enhance the stability, skin penetration, and therapeutic potential of CBD compared to non-stabilized formulations or CBD alone by leveraging the synergistic effects of its components. To test this hypothesis, the work is guided by specific objectives: to formulate and optimize the chitosan-stabilized nanoemulsion; to characterize the optimized system in terms of its key physicochemical properties and *in vitro* release profile; to assess its stability under various storage conditions; and to evaluate its *ex vivo* skin permeation and preliminary safety profile using suitable biological models. By systematically addressing these objectives, this research seeks to provide a novel delivery platform for targeting the multifaceted pathology of psoriasis.

## 2. Materials and Methods

### 2.1. Materials

Argan oil was cold-pressed from unroasted kernels (Setragal, Tangier, Morocco). Chitosan, obtained from pink shrimp shells (*Parapenaeus longirostris*, Larache, Morocco), had a degree of deacetylation (DDA) of 88.3 ± 0.5% (determined by potentiometric titration) and a viscosity-average molecular weight (Mv) of 409 ± 3 kDa. Cannabidiol crystals were extracted from CBD rich strains of *Cannabis Sativa* (CBD, ENDOCA^®^, Hoofddorp, The Netherlands). The following materials and chemicals were also used: Spectra/Por^®^ BioTech cellulose membrane (Spectrum Laboratories, Rancho Dominguez, CA, USA); pig skin (Compostelana de Carnes s.l, Santiago de Compostela, Spain); HaCaT cells (Cytion, Eppelheim, Germany); wild-type *Caenorhabditis elegans* (*C. elegans*) N2 and *Escherichia coli* (*E. coli*) OP50 (Caenorhabditis Genetics Center, University of Minnesota, Minneapolis, MN, USA); Milli-Q water (Millipore, Merck, Darmstadt, Germany); acetic acid, ethylenediaminetetraacetic acid (EDTA), formic acid, peptone from casein, and agar (Labkem, Dublin, Ireland); acetonitrile and methanol (Thermo Fisher Scientific, Loughborough, UK); dimethyl sulfoxide (DMSO, Merck, Darmstadt, Germany); Trypsin, Fetal Calf Serum (FCS), Eagle’s Minimum Essential Medium (EMEM), L-glutamine, non-essential amino acids, penicillin, streptomycin, and resazurin (Innoprot, Derio, Spain); Phosphate-Buffered Saline (PBS, Corning, Corning, NY, USA); Tocobiol^®^ C (a tocopherol/β-sitosterol/squalene blend) (BTSA, Madrid, Spain); Tween 80 (polysorbate 80) (Guinama, Valencia, Spain); Span 80 (Sorbitan monooleate) (Croda Inc., Snaith, UK). All chemicals and reagents were of analytical or High-Performance Liquid Chromatography (HPLC) grade and were used without further purification.

### 2.2. Nanoemulsions Formulation

#### 2.2.1. Formulation of NE and CS-NE

The argan oil nanoemulsions (NEs) were formulated using the Phase Inversion Composition (PIC) method, a low-energy emulsification technique. The aqueous phase, composed of Tween 80 and distilled water, was added dropwise to the lipid phase consisting of argan oil and Span 80. The mixture was then stirred for one hour at 1600 rpm. The concentrations of Span 80 and Tween 80 were adjusted to achieve a Hydrophilic-Lipophilic Balance (HLB) value of 11. The oil-to-surfactant weight ratio was varied (3:7, 4:6, 5:5, 6:4, 7:3), while the water content was maintained at 90% (*w*/*w*).

Chitosan-stabilized nanoemulsions (CS-NEs) were prepared by dissolving chitosan in 1% (*v*/*v*) acetic acid, followed by mixing the solution with NE at 1:1 (*w*/*w*) ratio. The mixture was stirred for one hour at 1600 rpm, resulting in a final chitosan concentration of 1% (*w*/*w*). The formulation was then sonicated for 2 min using a microtip probe (Sonopuls ultrasonic homogenizer, Bandelin, Berlin, Germany) at 20% amplitude with 5 s ON/10 s OFF pulse cycle. The probe was positioned at a depth corresponding to two-thirds of the formulation’s height.

#### 2.2.2. Formulation of CBD-NE and CBD-CS-NE

The CBD loaded nanoemulsions (CBD-NE) and chitosan-stabilized CBD nanoemulsions (CBD-CS-NE) were prepared using the same methods described previously. The lipid phase was prepared by dissolving CBD and Tocobiol^®^ C in argan oil. To ensure complete dissolution while minimizing thermal exposure, this mixture was warmed to 40 °C with continuous stirring for 30 min and visually inspected for clarity before use. The appropriate quantities were added to achieve a final concentration of 0.10% (*w*/*w*) for CBD and 0.05% (*w*/*w*) Tocobiol^®^ C in the final nanoemulsion.

### 2.3. Nanoemulsions Characterization

#### 2.3.1. pH, Droplet Size, PDI, and Zeta Potential

The pH value was measured at 25 °C using a Hanna pH meter. The average droplet size and polydispersity index (PDI), which indicates the uniformity of droplet sizes, were analyzed by Dynamic Light Scattering (DLS). The ζ-potential, a measure of the electrostatic repulsion between droplets that prevents aggregation, was determined by Electrophoretic Light Scattering (ELS). Both measurements were performed at 25 °C using a Malvern Zetasizer Ultra instrument, following 1:1000 dilution of the samples. All measurements were performed in triplicates.

#### 2.3.2. Viscosity Studies

Formulation viscosities were measured in triplicate at different temperatures (20 to 37 °C at 20 rpm) and shear rates (10 to 100 rpm at 20 °C) using a rotational viscometer (ViscoQC 300, Anton Paar, Graz, Austria).

#### 2.3.3. Stability Studies

Physical stability was assessed by storing the samples at 40 °C for 2 h, followed by centrifugation at 2655× *g* and 17,949× *g* for 30 min using an Eppendorf 5804 R Centrifuge (Eppendorf SE, Hamburg, Germany). Storage stability was evaluated by keeping samples at room temperature and monitoring their homogeneity, droplet size, PDI, and ζ-potential at regular intervals over 3 months.

#### 2.3.4. *In Vitro* Drug Release

The *in vitro* release of CBD from the formulations (CBD-NE and CBD-CS-NE) was studied using vertical Franz diffusion cells fitted with a Spectra/Por^®^ BioTech cellulose ester dialysis membrane (8–10 kDa molecular weight cut-off). Each membrane was trimmed and placed between the donor and receptor compartments of the Franz cell, ensuring a tight seal to prevent leakage. A 1 mL sample of each formulation was placed in the donor chamber, while receptor compartment was filled with 6.5 mL of receptor medium consisting of 1% (*w*/*v*) Tween 80 in PBS. The system was incubated at 36 °C under gentle orbital stirring at 100 rpm using a Unimax 1010 incubator (Heidolph Instruments, Schwabach, Germany). At specified time intervals (0.5, 1, 2, 3, 4, 5, 6, 7, 8 and 24 h), a 1 mL aliquot of the receptor medium was withdrawn and replaced with an equal volume of fresh medium to maintain constant volume. The drug concentration in each sample was determined using Ultra High-Performance Liquid Chromatography (UHPLC) with a ACQUITY UPLC H-Class Plus system equipped with a BEH C18 ACQUITY UPLC column (2.1 mm × 50 mm, 1.7 μm) (Waters Corporation, Milford, MA, USA). The mobile phases consisted of 0.1% (*v*/*v*) formic acid in acetonitrile and 0.1% (*v*/*v*) formic acid in water, used at 72.5% and 25.5%, respectively, with a flow rate of 0.5 mL/min. The acquisition time was 10 min, and the column was thermostatted at 35 °C. Sampling was automated using the Waters ACQUITY Sample Manager, with an injection volume of 2 μL and an injection time of 2 min. Standard solutions (0.05 to 100 μg/mL) were prepared by serial dilutions of a CBD methanolic stock solution (0.1 mg/mL) in methanol. The cumulative drug release per unit area was calculated and plotted as a function of time.

#### 2.3.5. *Ex Vivo* Skin Permeation

Skin permeation was studied using full-thickness porcine ear skin obtained by dermatome sectioning and mounted on Franz diffusion cells, following the same protocol as the *in vitro* release study. A 1 mL sample of each formulation (CBD-NE and CBD-CS-NE) was placed in the donor chamber, and the receptor chamber was filled with 6.5 mL of PBS containing 1% (*w*/*v*) Tween 80. The system was maintained at 36 °C with gentle stirring (100 rpm). At predetermined intervals (0.5 to 24 h), 1 mL samples were collected from the receptor compartment and replaced with fresh medium. CBD concentrations were quantified by UHPLC using a Waters ACQUITY UPLC system as described in the *In vitro* Drug Release section. The cumulative amount permeated per unit area was calculated and plotted over time. CBD permeation from pure argan oil, at the same concentration as the formulations, was used as a control. The steady-state flux (J) was determined from the slope of the linear portion of the cumulative permeation curve. The permeability coefficient (K_p_) and the enhancement ratio (ER) were calculated as follows [11]:

K_p_ = J/C_0_ (1)

ER (%) = J_test_/J_control_ (2)

where J is the steady-state flux (µg/cm^2^.h); C_0_ is the initial CBD concentration in the donor chamber (µg/mL).

#### 2.3.6. Drug Release Profile

Release kinetics were analyzed by fitting the initial linear phase of the cumulative release data from the *in vitro* drug release and *ex vivo* skin permeation studies to established mathematical models to elucidate the underlying release mechanisms. The zero-order, first-order, Higuchi, and Korsmeyer–Peppas models were applied [12]. The most appropriate model was identified based on linear regression analysis, using the adjusted coefficient of determination (R^2^) value as the selection criterion.

Zero-order model:

Q_t_ = Q_0_ + K_0_ × t (3)

First-order model:

ln (Q_t_) = ln (Q_0_) + K_0_ × t (4)

Higuchi model:

Q_t_ = K_H_ × √t (5)

Korsmeyer–Peppas model:

Q_t_ = K_HP_ × t^n^ (6)

where Q_0_ is the initial drug amount in the formulation; Q_t_ is the amount of drug released at time t; K_0_, K_1_ and K_H_ are release constants for respective models; K is the kinetic constant; n is the release exponent indicative of the transport mechanism.

#### 2.3.7. *In Vitro* Cytotoxicity

HaCaT cells (Cytion, Germany), an immortalized human keratinocyte cell line, were cultured under standard conditions at 37 °C in a humidified incubator with 5% CO_2_. The cells were subcultured one to two times per week to maintain healthy growth. For detachment, cells were treated with a trypsin/EDTA solution containing 0.025% trypsin and 0.02% EDTA. The enzymatic reaction was neutralized by adding an equal volume of complete culture medium supplemented with 15% FCS. The cell suspension was then centrifuged at 900 rpm for 5 min, and the resulting pellet was resuspended in fresh complete medium. The culture medium used was EMEM enriched with 10% FCS, 1% L-Glutamine (200 mM), 100× non-essential amino acids, and antibiotics (penicillin 100 µg/mL and streptomycin 100 µg/mL).

The cytotoxic potential of the test formulations was evaluated using the resazurin reduction assay in a 96-well plate format. HaCaT cells were seeded at a density of 125 µL per well of complete EMEM. After 24 h, an additional 100 µL of medium was added to each well. Then, 25 µL of the test formulations were added to the respective wells, namely NE, CBD-NE, CS-NE, and CBD-CS-NE, at CBD concentrations of 25 μM, 12.5 μM and 6.25 μM. The plate included control wells as follows: blank control wells contained only 125 µL of culture medium without cells; negative control wells contained untreated cells; and solvent control wells contained cells treated with DMSO, the solvent used for the test samples. Cells were exposed to the test formulations in quadruplicate for 24 h to assess cytotoxicity.

Following the exposure period, the culture medium was carefully removed from each well, and cells were gently washed with PBS to eliminate residual compounds. A resazurin working solution was freshly prepared in culture medium to a final concentration of 43.63 μM, and 100 µL was added to each well. Plates were then incubated for 4 h at 37 °C in a 5% CO_2_ atmosphere to allow for the metabolic reduction of resazurin to resorufin by viable cells. Fluorescence was measured using a FLUOstar OPTIMA microplate reader (BMG LABTECH, Ortenberg, Germany) with excitation at 530 nm and emission at 590 nm. Cell viability (%) was calculated using the following formula:

Cell viability (%) = (N_T_/N_C_) × 100 (7)

where N_T_ is the number of viable cells in the treatment group and N_C_ is the number of viable cells in the control group.

This allowed for the quantification of cell viability and the evaluation of cytotoxic effects induced by the tested formulations.

#### 2.3.8. *In Vivo* Toxicity in *Caenorhabditis elegans* Model

Wild-type *C. elegans* N2 strains were routinely maintained on solid Nematode Growth Medium (NGM) supplemented with *E. coli* OP50 as a food source. The cultures were kept at a constant temperature of 20 °C in a Biochemical Oxygen Demand (BOD) incubator. To generate age-synchronized worm populations, a sodium hypochlorite treatment was applied, followed by the isolation of eggs laid overnight in chloride medium (84 mM NaCl). These eggs were then used for experimental procedures.

Approximately 20 young *C. elegans* (L1 larval stage) from the N2 strain were transferred into Eppendorf^®^ tubes containing liquid chloride medium (85 mM NaCl). The worms were exposed to various concentrations of test samples, along with a vehicle control (negative control), and incubated at 20 °C with continuous shaking for 30 min. The test samples included CBD dissolved in DMSO, CBD-NE, and CBD-CS-NE, at CBD concentrations of 25 μM, 50 μM, and 100 μM. Additionally, NE, CS-NE, and DMSO were tested at concentrations equivalent to 100 μM of CBD. Following exposure, the worms were rinsed three times with chloride medium and transferred to 24-well plates. After 24 h, worm viability was assessed by categorizing individuals as either alive or dead if they exhibited no movement of the pharynx or body. The experiment was conducted in triplicate across three independent experimental groups. The survival percentage was calculated using the following formula:

Survival (%) = (N_f_/N_i_) × 100 (8)

where N_f_ is the number of worms alive at the end of the experiment and N_i_ is the number of worms alive at the start of the experiment.

### 2.4. Statistical Analysis

Data are presented as mean ± standard deviation, with the mean rounded to the same number of decimal places as the standard deviation. Statistical significance was determined by analysis of variance (ANOVA) with Tukey’s multiple comparisons test (*p* < 0.05). All analyses were conducted using GraphPad Prism 8.0.2.

## 3. Results

### 3.1. Formulation Composition

Stable nanoemulsion formulations (NE) were identified by varying the ratio of argan oil to the surfactant mixture. The proportions of Span 80 and Tween 80 within the surfactant mixture (SM) were adjusted to achieve a HLB value of 11.0, which is reported as optimal for argan oil [13].

**Figure 1:**
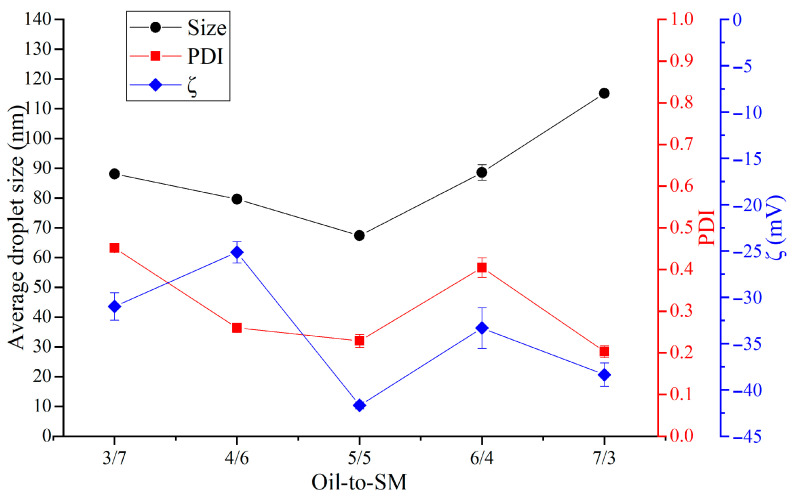
Effect of oil-to-surfactant mixture ratio on average droplet size, PDI, and ζ-potential of the nanoemulsions (NE).

The droplet size results (Figure 1) showed a complex relationship with the oil-to-SM ratio. The smallest droplet size (67.4 ± 0.5 nm) was achieved at a 5:5 ratio, indicating an optimal balance between oil and surfactant that likely promotes efficient droplet formation. In contrast, the largest droplets (115.2 ± 0.4 nm) were observed at a 7:3 ratio, suggesting that higher oil content can lead to the formation of larger droplets. Interestingly, the 6:4 and 3:7 ratios produced similar droplet sizes (89 ± 3 nm and 88.1 ± 0.6 nm, respectively), highlighting a non-linear trend in size changes relative to the oil-to-SM ratio.

The PDI, which reflects droplet size uniformity, decreased as the oil content increased (Figure 1). The highest PDI (0.452 ± 0.008) was recorded at the 3:7 ratio, suggesting less uniformity. Conversely, the lowest PDI values (0.229 ± 0.016 and 0.203 ± 0.014) were observed at the 5:5 and 7:3 ratios, respectively, indicating greater uniformity. These ratios offer a good balance between droplet size optimization and uniformity, making them promising formulations.

ζ-potential values of the NEs were negative (Figure 1). Argan oil has a high unsaturated fatty acid content (85–90%), mainly oleic and linoleic acids, which confer a negative surface charge to the oil droplets (Figure 1). The ζ-potential value of NEs was influenced by the proportion of oil in the mixture. An increase in the proportion of surfactants relative to oil reduced the magnitude of the ζ-potential. The two surfactants used in the preparation of the NEs do not possess ionizable groups; therefore, increasing their concentration and decreasing the proportion of oil induces a shielding of surface charges, reducing the ζ-potential value. The ζ-potential values obtained with the highest proportions of oil suggest strong electrostatic repulsion between droplets, which prevents aggregation and ensures stability. In contrast, the lowest ζ-potential (−25.14 ± 1.15 mV), indicating reduced stability, was found at the 4:6 ratio. Interestingly, while the 7:3 ratio, despite producing the largest droplets, maintained a relatively high stability with a ζ-potential of −38.4 ± 1.3 mV, suggesting good resistance to aggregation [14].

Overall, the 5:5 oil-to-SM ratio emerged as the most balanced formulation, producing the smallest droplet size, a relatively low PDI, and high colloidal stability. In contrast, formulations with higher oil content, such as 7:3 ratio, yielded larger droplet sizes but demonstrated better uniformity and relatively strong stability, making them suitable for applications requiring higher oil loading.

These results highlight the impact of oil-to-SM ratio on droplet size, PDI, and ζ-potential—critical parameters in assessing nanoemulsion stability and uniformity—underscoring the importance of optimizing this ratio for effective dermal drug delivery [15].

The final compositions (*w*/*w*%) of the four selected nanoemulsions were as follows. The plain nanoemulsion (NE) contained 5.0% argan oil, 5.0% surfactant mixture (Tween 80:Span 80, 1:1 (*w*/*w*)), and 90.0% water. The chitosan-stabilized nanoemulsion (CS-NE) contained 2.46% oil, 2.46% surfactants, 1.0% chitosan, 0.49% acetic acid (from the chitosan stock solution), and 93.58% water. The CBD-loaded nanoemulsion (CBD-NE) contained 4.99% oil, 4.99% surfactants, 0.10% CBD, 0.05% Tocobiol^®^ C, and 89.87% water. The chitosan-stabilized CBD-loaded nanoemulsion (CBD-CS-NE) contained 2.46% oil, 2.46% surfactants, 1.0% chitosan, 0.49% acetic acid, 0.10% CBD, 0.05% Tocobiol^®^ C, and 93.43% water.

The 0.1% (*w*/*w*) CBD concentration employed in this study was selected based on prior evidence demonstrating its therapeutic efficacy in inflammatory skin conditions. Preclinical studies have shown that topical 0.1% CBD significantly reduces psoriasis-like inflammation, epidermal thickness, and hyperproliferation in murine models [16]. Furthermore, clinical application of 0.1% CBD has been reported to accelerate healing and reduce lesion size in inflammatory mucosal conditions without adverse effects [17], supporting its suitability for dermal delivery in the present formulation study.

### 3.2. pH, Droplet Size, PDI, and Zeta Potential

Upon preparation, all nanoemulsions formed homogeneous, white dispersions. The plain formulations (NE and CBD-NE) were opaque, milky-white liquids with low viscosity and rapid flow. In contrast, the chitosan-stabilized formulations (CS-NE and CBD-CS-NE) appeared less opaque, with a translucent, pearlescent quality and a noticeably higher, gel-like viscosity.

**Figure 2:**
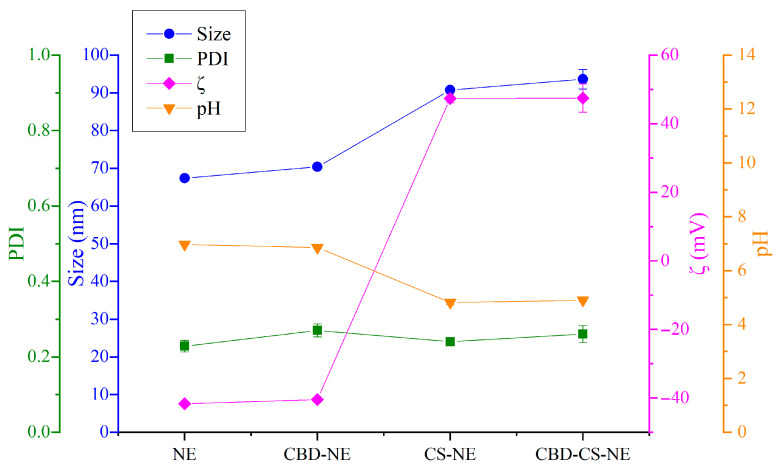
Variation of average droplet size, PDI, ζ-potential, and pH as a function of formulation composition.

Further characterization (Figure 2) showed that NE had the smallest droplet size (67.4 ± 0.5 nm), indicating a compact and efficient formulation. The inclusion of chitosan in CS-NE significantly increased the droplet size to 90.7 ± 0.6 nm, likely due to droplet coating or aggregation effects. However, CBD addition did not statistically influence the droplet size for either formulation, with or without chitosan. The CBD-CS-NE formulation, combining both CBD and chitosan, had the largest droplet size (94 ± 3 nm).

As shown in Figure 2, the PDI results indicated uniform droplet size distributions, with no statistical differences between the formulations. A low PDI signifies a highly uniform droplet population, a key factor for physical stability and predictable drug release.

ζ-potential analysis of CBD-NE, CS-NE and CBD-CS-NE, revealed differences among the formulations. The base NE showed the highest stability, with a ζ-potential of −41.7 ± 0.4 mV (Figure 2), while other formulations varied depending on their composition. CBD-NE exhibited a comparable value (−41.0 ± 0.6 mV), indicating that the addition of CBD did not significantly alter surface charge. In contrast, chitosan-containing formulations (CS-NE and CBD-CS-NE) displayed highly positive ζ-potential (47.4 ± 1.3 mV and 47 ± 4 mV, respectively), with no significant differences caused by CBD addition. These positive values are consistent with the cationic nature of chitosan, which interacts with and adsorbs onto the negatively charged droplet surfaces [9].

The pH values also varied among formulations (Figure 2). NE and CBD-NE exhibited near-neutral pH values (6.97 ± 0.01 and 6.86 ± 0.03, respectively), consistent with their base components. In contrast, CS-NE and CBD-CS-NE showed acidic pH values (4.82 ± 0.09 and 4.90 ± 0.05, respectively), reflecting the effect of the acetic acid used to dissolve the chitosan. The reduction in the pH of these formulations may also contribute to the neutralization of fatty acids and, consequently, to the changes in ζ-potential observed for CS-NE and CBD-CS-NE. Moreover, the addition of CBD to NE and CS-NE did not significantly alter the formulation pH values.

In summary, these results demonstrate how compositional adjustments influence key physicochemical properties, informing formulation strategies for stable and effective nanoemulsions. The NE formulation exhibited the smallest droplet size, the most uniform distribution, high colloidal stability, and near-neutral pH, making it a highly stable and consistent base. The addition of CBD in CBD-NE maintained these favorable characteristics with minimal changes, resulting in a slight increase in droplet size while preserving overall stability. Formulations containing chitosan (CS-NE and CBD-CS-NE) significantly altered physical properties, producing larger droplets, positive ζ-potential, and slightly acidic pH values. Nonetheless, their relatively low PDI values indicate that the droplet size distribution remained fairly uniform.

These findings highlight the distinct effects of chitosan on formulation characteristics, supporting their potential customization for dermal and psoriasis-related applications. The small size of chitosan-coated nanoemulsions is favorable for dermal application, as smaller droplets typically enhance drug penetration and spreadability, thereby improving delivery to the target site. The low PDI indicates a highly uniform droplet population, which is a key factor for physical stability and predictable drug release. Additionally, the strong positive surface charge promotes electrostatic repulsion between droplets, preventing aggregation and enhancing colloidal stability during storage. This positive charge may also promote bioadhesion and stronger interactions with negatively charged skin components, which can improve local drug retention [18,19]. Human skin typically has a pH between 4.5 and 6.0, and the optimal physiological range for dermal applications spans 4.0 to 7.0 [20]. All tested formulations fell within this range, suggesting good skin compatibility and low irritation potential. Furthermore, the chitosan coating may help prevent premature drug release and improve retention at the application site, leading to prolonged therapeutic effects [18]. As a biocompatible polymer with anti-inflammatory properties [21], chitosan may complement the therapeutic action of encapsulated drugs particularly in the treatment of inflammatory skin disorders such as psoriasis.

### 3.3. Viscosity Analysis

**Figure 3:**
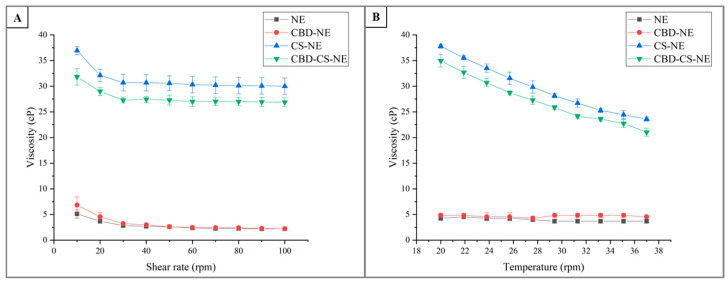
Viscosity as a function of shear rate (**A**) and temperature (**B**) for the formulations NE, CBD-NE, CS-NE, and CBD-CS-NE.

Viscosity is a critical parameter for evaluating the flow properties and stability of formulations, influencing both their processing and application. The viscosity profiles of the different formulations were measured at shear rates ranging from 10 to 100 rpm at 25 °C (Figure 3A). The NE and CBD-NE formulations demonstrated low viscosities and followed a similar trend, starting at 5.1 ± 0.8 cP and 6.8 ± 1.6 cP at 10 rpm, respectively, and gradually decreasing to 2.22 ± 0.08 cP for both at 100 rpm. The reduction in viscosity with increasing shear rate indicated shear-thinning behavior, wherein the internal structure of the formulation is disrupted under shear forces, facilitating flow [22].

In contrast, formulations containing chitosan (CS-NE and CBD-CS-NE) exhibited substantially higher viscosities across all shear rates compared to their non-chitosan counterparts. At 10 rpm, the viscosities were 37.0 ± 0.8 cP and 31.8 ± 1.6 cP, decreasing steadily to 30.0 ± 1.6 cP and 26.9 ± 0.9 cP at 100 rpm, respectively. The elevated viscosity is attributed to the presence of chitosan, a polymer known for its thickening properties. The progressive decrease in viscosity with increasing shear rate confirms the shear-thinning behavior typical of polymeric systems, where molecular entanglements and interactions are reduced under shear [23].

NE and CBD-NE showed no significant difference in viscosity, suggesting that the incorporation of CBD did not markedly affect the rheological properties of the nanoemulsion matrix. However, in the presence of chitosan, the viscosity values differed significantly, indicating that the inclusion of CBD introduced structural complexity that resulted in moderated viscosity.

The temperature-dependent viscosity data (Figure 3B) provided additional insights into the thermal behavior of the formulations between 20 °C and 37 °C. The NE formulation maintained consistently low viscosities, starting at 4.3 ± 0.4 cP at 20 °C, decreasing slightly to 3.7 ± 0.4 cP, and then stabilizing. This minor variation suggests that NE maintained stable flow properties over the studied temperature range, with minimal sensitivity to thermal changes.

CBD-NE also exhibited minimal thermal sensitivity, with viscosities ranging from 4.8 ± 0.4 cP to 4.548 ± 0.000 cP. Notably, the addition of CBD did not significantly alter the thermal behavior of the formulation.

CS-NE exhibited higher viscosities across all temperatures, starting at 37.8 ± 0.4 cP at 20 °C and gradually decreasing to 23.6 ± 0.4 cP at 37 °C. This decline reflects the temperature sensitivity of chitosan, as increasing thermal energy reduces molecular entanglements and thickening interactions—a typical characteristic of polymer-based systems [23].

Although CBD-CS-NE showed viscosities similar to CS-NE, they were consistently lower and significantly different. Beginning at 35.0 ± 1.2 cP at 20 °C, the viscosity decreased to 21.0 ± 0.8 cP at 37 °C. This trend mirrored the thermal sensitivity of CS-NE but suggested that CBD may moderate the temperature-induced viscosity drop, possibly by influencing the chitosan’s intermolecular interactions.

The viscosity study revealed clear distinctions between the formulations. NE and CBD-NE, with the lowest viscosities, were highly flowable, making them ideal for easy application, rapid dispersion, and exhibited minimal temperature sensitivity. Their pronounced shear-thinning behavior and stable viscosity across a wide temperature range suggest good storage stability and ease of use under mechanical stress.

In contrast, CS-NE and CBD-CS-NE, with significantly higher viscosities, are robust systems suitable for bioadhesive and controlled-release applications [24]. The inclusion of chitosan introduced considerable resistance to flow, which is expected to enhance film-forming ability and residence time on the skin, reduces runoff, and provide a desirable sensory feel upon application. However, their viscosities decreased markedly with rising temperatures, reflecting the polymeric nature of chitosan and underscoring the importance of storage temperature control for these systems. An interesting empirical observation was the slightly lower viscosity of CBD-CS-NE compared to the CS-NE formulation. While the exact cause of this difference cannot be determined from rheological data alone, it indicates a subtle interaction within the formulation. Future studies employing structural analysis techniques would be necessary to elucidate whether this results from a direct molecular interaction between CBD and chitosan, a modification of the droplet interface, or another formulation effect.

The observed shear-thinning and temperature-dependent behavior further underscore the unique characteristics of these formulations. Shear-thinning is advantageous in pharmaceutical applications, as a high apparent viscosity at low shear limits the movement of the dispersed phase, stabilizing the system during storage, while low viscosity at high shear ensures good spreadability and ease of application [12].

### 3.4. Stability Studies

In the centrifugation test, all formulations demonstrated remarkable physical stability, showing no phase separation when subjected to centrifugation at 2655× *g* for 30 min. Notably, the chitosan-stabilized nanoemulsions maintained their integrity even at higher centrifugation speeds of up to 17,949× *g* at 40 °C, underscoring the robust stabilizing effect of chitosan on the nanoemulsion system.

**Figure 4:**
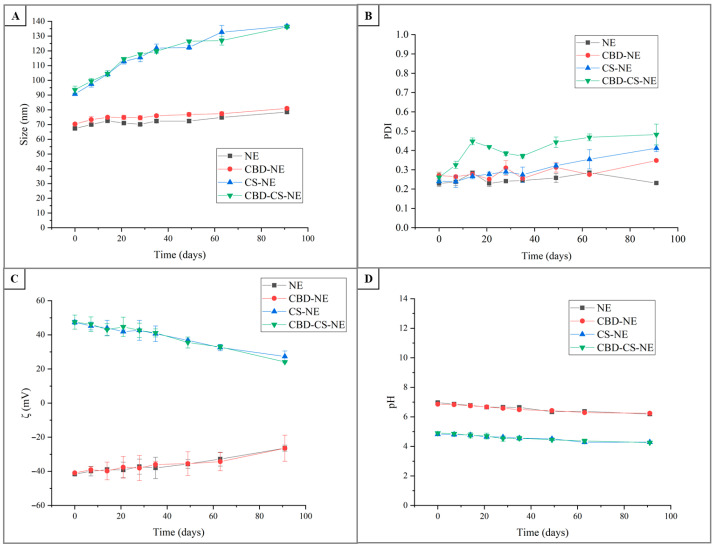
Time-dependent variations in (**A**) droplet size, (**B**) PDI, (**C**) ζ-potential, and (**D**) pH for the formulations NE, CBD-NE, CS-NE, and CBD-CS-NE.

The storage stability study assessed changes in homogeneity, droplet size, PDI, ζ-potential, and pH over 91 days (Figure 4). These parameters are critical for evaluating the long-term stability of the formulations, particularly in terms of resistance to aggregation, uniformity, and colloidal stability. All formulations remained physically stable, retaining a homogeneous single-phase structure throughout the study period.

The droplet size of NE gradually increased from 67.4 ± 0.5 nm on day 0 to 78.5 ± 1.0 nm on day 91, indicating a slight degree of destabilization over time. This increase was relatively minor, suggesting good overall physical stability. CBD-NE followed a similar trend, with droplet size increasing from 70.4 ± 0.6 nm to 81.0 ± 1.0 nm, reflecting slightly more pronounced destabilization than NE. CS-NE exhibited a more marked increase, from 90.7 ± 0.6 nm to 136.7 ± 1.2 nm, likely due to the polymeric nature of chitosan which may promote aggregation. CBD-CS-NE showed a comparable pattern, with size increasing from 94 ± 3 nm to 136.3 ± 1.2 nm. Notably, droplet size was primarily influenced by the presence of chitosan, and CBD incorporation did not induce significant structural changes in terms of size.

The PDI of NE rose slightly from 0.229 ± 0.016 to 0.285 ± 0.005, indicating a small reduction in uniformity. CBD-NE exhibited a more notable increase from 0.270 ± 0.017 to 0.348 ± 0.006, suggesting increased heterogeneity in particle size distribution. CS-NE showed a significant rise in PDI from 0.240 ± 0.010 to 0.411 ± 0.017, while CBD-CS-NE increased markedly from 0.26 ± 0.02 to 0.48 ± 0.05, reflecting a pronounced loss of uniformity. Importantly, the PDI values between NE and CBD-NE, and between NE and CS-NE were not significantly different, indicating a comparable degree of formulation uniformity among these variants, regardless of the inclusion of cannabidiol or chitosan.

NE exhibited a decline in ζ-potential from −41.7 ± 0.4 mV to −26.4 ± 1.8 mV, indicating a reduction in surface charge and electrostatic stabilization. This reduction may be attributed to chemical and physical changes occurring during storage, particularly within the oil phase and surfactant layer. Argan oil contains free fatty acids, phenolics, tocopherols and other polar compounds that, along with their oxidation or hydrolysis products, may migrate toward the droplet interface and partially neutralize or mask negatively charged sites, thereby lowering the measured ζ-potential. Additionally, gradual degradation or rearrangement of surfactant molecules may generate or expose new interfacial species that alter the electrical double layer, contributing to the observed decrease in surface charge [25]. CBD-NE showed a similar trend, decreasing from −41.0 ± 0.6 mV to −26 ± 8 mV. In both cases, the reduction remained within an acceptable range, supporting adequate colloidal stability. CS-NE showed a decrease in ζ-potential from +47.4 ± 1.3 mV to +27 ± 3 mV, and CBD-CS-NE dropped from +47 ± 4 mV to +24.1 ± 0.5 mV, indicating reduced electrostatic repulsion. The similarity between NE and CBD-NE suggested that CBD incorporation did not affect the surface charge of nanoemulsion. Similarly, the comparable ζ-potential of CS-NE and CBD-CS-NE indicated that chitosan governs the electrostatic properties, and the presence of CBD did not significantly alter this behavior.

The pH of NE declined from 6.97 ± 0.01 on day 0 to 6.19 ± 0.08 on day 91, remaining within a stable range and suggesting minimal degradation. CBD-NE followed a similar pattern, decreasing from 6.86 ± 0.03 to 6.24 ± 0.05. Due to the presence of chitosan solubilized in acetic acid, CS-NE had a lower initial pH of 4.82 ± 0.09, which declined to 4.29 ± 0.06. CBD-CS-NE started at 4.90 ± 0.05 and decreased to 4.26 ± 0.05. These lower values reflected formulation characteristics rather than instability. The pH values of NE and CBD-NE, and those of CS-NE and CBD-CS-NE, were not significantly different. This consistency indicated that CBD inclusion did not substantially affect the acidity or alkalinity of the system. Therefore, the observed pH was largely governed by the base formulation components (with or without chitosan), and CBD did not introduce significant chemical changes that would alter pH.

Collectively, these results suggest that the addition of CBD did not significantly alter the physical or colloidal stability of the formulations within each type (with or without chitosan), reinforcing the compatibility of CBD with the nanoemulsion matrix. The stability data revealed a clear distinction between the plain and chitosan-stabilized systems. The NE and CBD-NE formulations maintained relatively stable droplet size, PDI, ζ-potential, and pH over 91 days, indicating good potential for long-term storage with minimal physical degradation. In contrast, while the CS-NE and CBD-CS-NE formulations provided initial stability and desirable functional properties (positive charge, increased viscosity), their long-term physical stability was more challenging. These formulations exhibited more substantial increases in droplet size and PDI over time compared to their non-chitosan counterparts. This suggests that the presence of chitosan, while beneficial for bioadhesion and initial colloidal charge, may introduce long-term instability that requires further optimization.

While this study assessed the physical and colloidal stability of the carrier systems, it is important to note a key limitation: the stability assessment did not include a chemical assay to quantify the integrity of the encapsulated CBD. Therefore, these data establish the physical shelf-life trends of the systems but do not confirm the chemical shelf-life of the active pharmaceutical ingredient. To advance the formulation toward pharmaceutical application, future studies must incorporate analytical methods, such as HPLC, to monitor CBD content and identify potential degradation products under various storage conditions.

Overall, the findings highlight a key formulation trade-off: chitosan imparts functional advantages relevant to dermal delivery, like the positive charge and bioadhesion but may compromise long-term physical stability compared to simpler nanoemulsions. All formulations remained within acceptable parameters, confirming their overall stability throughout the study period.

### 3.5. In Vitro Drug Release

**Figure 5:**
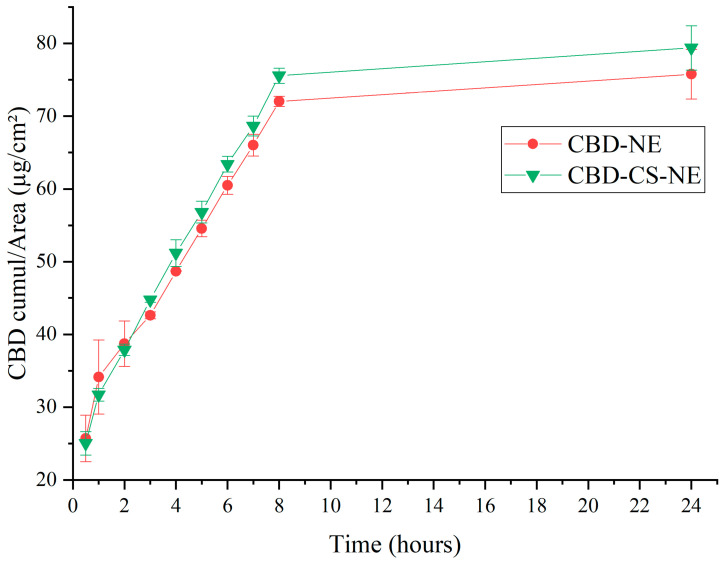
Cumulative CBD release per unit surface area (µg/cm^2^) over time for the CBD-NE and CBD-CS-NE formulations.

This *in vitro* study assessed the release profile of CBD cross a synthetic membrane. The cumulative release data demonstrated that CBD-NE and CBD-CS-NE exhibited comparable release profiles over time, with CBD-CS-NE consistently showing a higher cumulative release per unit area than CBD-NE (Figure 5). At 0.5 h, CBD-NE released 26 ± 3 µg/cm^2^, while CBD-CS-NE released 25.0 ± 1.6 µg/cm^2^, indicating similar initial diffusion rates. As the experiment progressed, CBD-CS-NE achieved a cumulative release per unit area of 79 ± 3 µg/cm^2^ after 24 h, compared to 76 ± 4 µg/cm^2^ for CBD-NE.

Both CBD-NE and CBD-CS-NE exhibited a sustained release profile over a 12 h period, with no abrupt fluctuations in the release rate, indicating their suitability for controlled CBD delivery.

Mathematical modeling was applied to assess the drug release behaviors of the formulations. The linear portion of the cumulative drug release per unit area over time was fitted to three kinetic models—Zero-Order, First-Order, Higuchi and Korsmeyer–Peppas—to identify the best-fit model describing CBD release and permeation mechanisms. Adjusted R^2^ values were as indicators of model suitability.

Although the cumulative release profiles of both CBD-NE and CBD-CS-NE exhibited near-linear behavior over the initial portion, resulting in high adjusted R^2^ values for the zero-order model (98.94% and 99.47%, respectively), such correlations are commonly observed for cumulative release data and should not be interpreted as definitive evidence of a true zero-order release mechanism. To gain further insight into the release mechanism, the data were also evaluated using diffusion-based and semi-empirical models [12]. Both formulations showed high correlation with the Higuchi model (adjusted R^2^ = 97.13% for CBD-NE and 98.46% for CBD-CS-NE), suggesting that diffusion plays a significant role in CBD release. The Korsmeyer–Peppas model yielded release exponent (n) values of 0.3520 for CBD-NE and 0.3901 for CBD-CS-NE. These n values (<0.45) are characteristic of Fickian diffusion-controlled release, indicating that CBD transport is primarily governed by diffusion through the oil–water interface of the nanoemulsion droplets and across the dialysis membrane [26].

It is important to note that due to the absence of sampling time points between 8 and 24 h, the current data cannot definitively confirm whether a plateau phase or a secondary release mechanism occurs during this interval.

Overall, the results indicate that while the release profiles may approximate zero-order behavior over the studied timeframe, the dominant release mechanism for both nanoemulsion systems is diffusion-controlled. The observed near-linear cumulative release is therefore attributed to sustained diffusion from the dispersed oil droplets under sink conditions, rather than to a concentration-independent zero-order process.

### 3.6. Ex Vivo Skin Permeation

**Figure 6:**
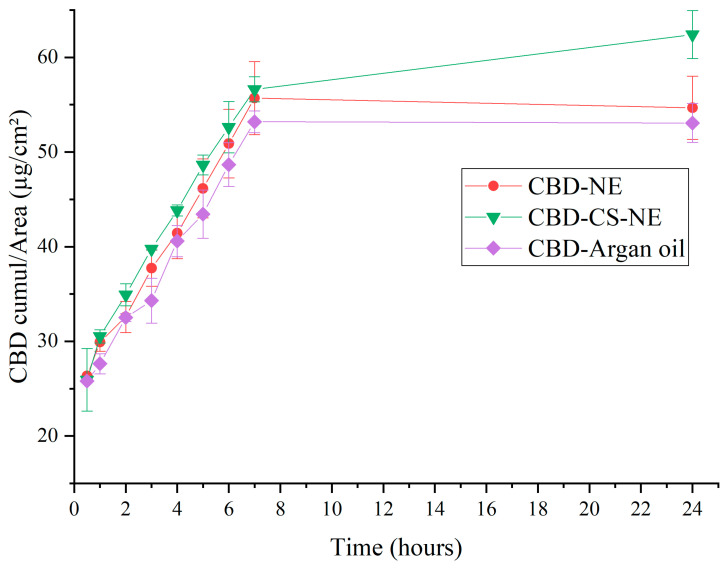
Cumulative skin permeation of CBD over 24 h from the CBD-NE, CBD-CS-NE, and CBD in argan oil formulations.

This *ex vivo* skin permeation study evaluated the cumulative permeation of CBD over 24 h from the two nanoemulsion formulations, CBD-NE and CBD-CS-NE, as well as from the free drug in oil (CBD-Argan oil) (Figure 6). The aim was to assess the extent of CBD penetration across the skin barrier, and to compare the effectiveness of the carrier systems.

The three formulations exhibited a gradual and continuous increase in CBD permeation, reaching 55 ± 3 µg/cm^2^, 62 ± 3 µg/cm^2^ and 53 ± 2 µg/cm^2^ for CBD-NE, CBD-CS-NE and CBD-Argan oil, respectively, at 24 h. The steady-state fluxes of the formulations were 4.4 ± 0.2 µg/cm^2^.h (CBD-NE), 4.58 ± 0.15 µg/cm^2^.h (CBD-CS-NE), and 4.18 ± 0.15 µg/cm^2^.h (CBD-Argan oil). The corresponding permeability coefficients were 0.00440 cm/h, 0.00458 cm/h, and 0.00418 cm/h (C_0_ = 1 mg/mL). Relative to CBD in argan oil, CBD-NE and CBD-CS-NE exhibited enhancement ratios of 1.05 and 1.10, respectively, representing improvements of 5.4% and 9.7%. Additionally, the flux of CBD-CS-NE was 4.1% higher than that of CBD-NE (enhancement ratio = 1.04).

Although the cumulative amount of CBD permeated after 24 h differed significantly among CBD-NE, CBD-CS-NE and CBD-Argan oil formulations (*p* < 0.05), the steady-state flux values were not statistically different. This indicates that once steady-state conditions were established, the intrinsic diffusion rate of CBD across the skin barrier was comparable for all formulations. Consequently, the observed enhancements for the nanoemulsion systems primarily affected the extent of permeation rather than the steady-state permeation velocity. The increased cumulative permeation from CBD-NE and CBD-CS-NE may be attributed to improved drug solubilization, increased thermodynamic activity at the skin interface, enhanced partitioning into the stratum corneum, and/or temporary drug retention within the skin layers [27]. In particular, chitosan functionalization produced the greatest increase in cumulative permeation, likely due to enhanced droplet–skin interactions and prolonged residence time at the skin surface, despite having no measurable effect on steady-state flux [28]. This interpretation is subject to the limitation that the relative contribution of chitosan versus the nanoemulsion matrix to enhanced permeation cannot be fully decoupled without comparative studies using a hydrophilic model drug.

Kinetic modeling of the *ex vivo* permeation data showed high correlations with both the zero-order (R^2^ ≈ 99%) and Higuchi models (R^2^ ≈ 98–99%) for all formulations. While the near-linear cumulative permeation profiles may approximate zero-order behavior over the investigated time period, such high correlations are characteristic of steady-state diffusion through skin and should not be interpreted as definitive evidence of a true zero-order mechanism. To further elucidate the permeation mechanism, the Korsmeyer–Peppas model was applied. The release exponent (n) values for CBD-NE (0.2754), CBD-CS-NE (0.2908), and CBD-Argan oil (0.2710) were all below 0.45, indicating Fickian diffusion–controlled permeation. This finding is consistent with diffusion-driven transport across the skin barrier under steady-state conditions and supports the Higuchi-based interpretation.

Overall, the kinetic and permeation analyses confirm that CBD transport across the skin from all formulations is predominantly governed by Fickian diffusion. The key practical finding is not a difference in the steady-state diffusion rate, but a significant enhancement in the total drug delivered by the nanoemulsion systems, particularly the chitosan-stabilized formulation (CBD-CS-NE). This has direct therapeutic implications: a greater cumulative permeation suggests that more active ingredient can reach the target sites in the skin over a standard dosing period, potentially improving efficacy. Furthermore, the chitosan-functionalized nanoemulsion offers additional practical advantages essential for a topical product: its positive charge and increased viscosity promote bioadhesion and extended residence time on the psoriatic skin, which may improve patient compliance and local bioavailability. Therefore, while the fundamental permeability of the skin to CBD remains constant, the chitosan-stabilized nanoemulsion represents a superior delivery strategy. It enhances the extent of delivery, provides a stable and protective carrier for the hydrophobic drug, and incorporates features conducive to topical application, together addressing key formulation challenges for treating chronic skin conditions like psoriasis.

### 3.7. In Vitro Cytotoxicity

**Figure 7:**
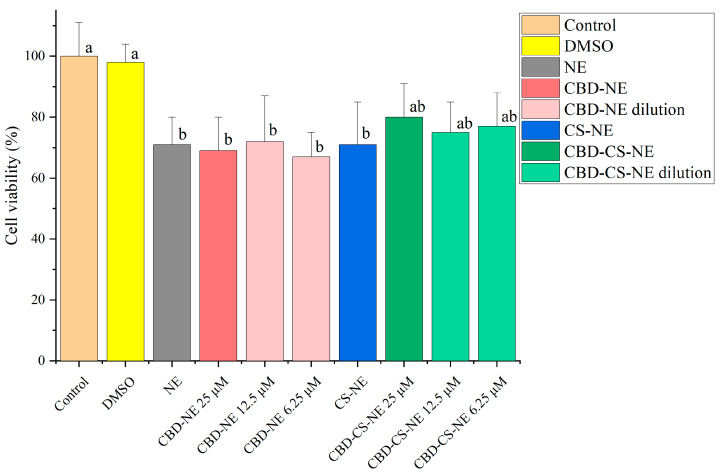
Viability of HaCaT cells under control conditions and following exposure to DMSO, NE, CBD-NE (25 μM, 12.5 μM, 6.25 μM), CS-NE, and CBD-CS-NE (25 μM, 12.5 μM, 6.25 μM). Different lowercase letters denote statistically significant differences between groups (Tukey’s post hoc test, *p* < 0.05).

The formulations NE, CBD-NE, CS-NE, and CBD-CS-NE were evaluated for their cytotoxicity on HaCaT keratinocyte cells (Figure 7).

The control group showed a standard deviation of ±11%, demonstrating the variability of the untreated cells, while the DMSO control exhibited a cell viability of 98 ± 6%, confirming the non-toxicity of the solvent.

The NE and CS-NE formulations exhibited cell viabilities of 71 ± 9% and 71 ± 14%, respectively, indicating weak cytotoxicity and overall good compatibility with HaCaT cells. The CBD-NE formulation showed similar viability values (69 ± 11%, 72 ± 15%, and 67 ± 8%) at CBD concentrations of 25 μM, 12.5 μM, and 6.25 μM, respectively. These comparable results suggest that the individual addition of either chitosan or CBD did not significantly alter the cytotoxic profile of the NE formulation.

The CBD-CS-NE formulation containing 25 μM of CBD demonstrated a higher cell viability of 80 ± 11%, falling within the non-cytotoxic range. Upon dilution, cell viability remained stable: 75 ± 10% at 12.5 μM and 77 ± 11% at 6.25 μM, indicating that concentration had no significant impact on cytotoxicity.

The study demonstrated that all tested formulations maintained acceptable levels of cell viability, with no substantial cytotoxicity observed. According to ISO 10993-5:2009 standard [29], cell viability above 80% is classified as non-cytotoxic, 80–60% as weak cytotoxicity, 60–40% as moderate, and below 40% as strong cytotoxicity [30].

The NE, CS-NE, and CBD-NE formulations exhibited weak cytotoxicity, with statistically comparable viability values. This indicates that the incorporation of chitosan or CBD did not significantly affect cytotoxicity. Additionally, no notable variations were observed between undiluted and diluted CBD-containing samples, suggesting consistent cytotoxicity profiles across concentrations.

The moderate cytotoxicity noted for CS-NE may be attributed to chitosan’s molecular-weight-dependent pro-apoptotic activity and its electrostatic interactions with negatively charged cell membranes, which can moderately compromise cell viability [31]. In contrast, the presence of CBD in the CBD-CS-NE formulation appears to mitigate these effects, likely through a counterbalancing or neutralizing interaction that reduces cytotoxicity [32].

Among all formulations, CBD-CS-NE exhibited the highest cell viability at all tested concentrations, falling within the non-cytotoxic range. This suggests a synergistic effect between CBD and chitosan that enhances overall biocompatibility, making this formulation particularly advantageous for dermatological applications requiring high cell compatibility.

In conclusion, while all formulations demonstrated mild to moderate cytotoxicity, none showed drastic reductions in HaCaT keratinocyte viability. CBD-CS-NE emerged as the most biocompatible formulation, underscoring its potential for safe and effective use in skin-related therapies where cellular compatibility is essential.

### 3.8. In Vivo Toxicity in Caenorhabditis elegans Model

**Figure 8:**
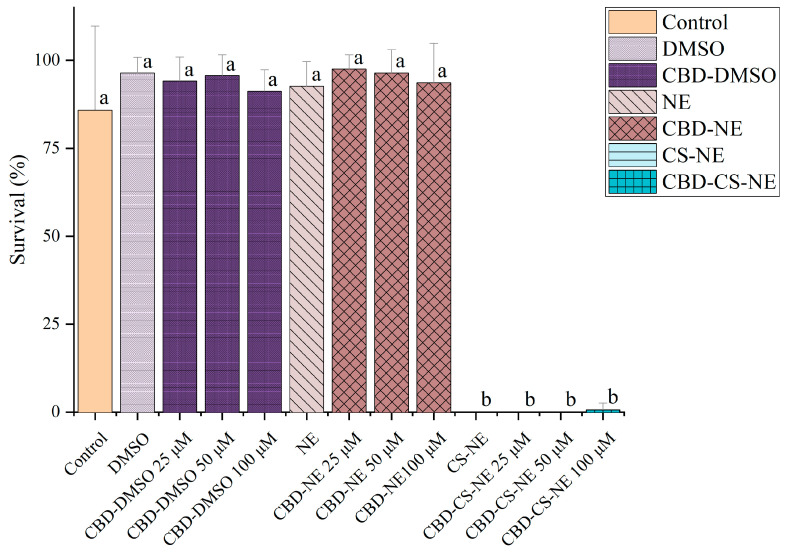
Survival of *C. elegans* under control conditions and following exposure to DMSO, CBD-DMSO (25 μM, 50 μM, 100 μM), NE, CBD-NE (25 μM, 50 μM, 100 μM), CS-NE, and CBD-CS-NE (25 μM, 50 μM, 100 μM). Different lowercase letters denote statistically significant differences between groups (Tukey’s post hoc test, *p* < 0.05).

This *in vivo* toxicity study assessed the survivability of *C. elegans* following exposure to various CBD-containing formulations at different concentrations. The survival rates of the nematodes were quantified under different experimental conditions (Figure 8).

The control group exhibited a survival rate of 86 ± 24%, indicating the baseline viability of the untreated *C. elegans*. The relatively high standard deviation suggested notable variability within the group, likely due to inherent biological differences among the nematodes.

Treatment with DMSO and CBD in DMSO (CBD-DMSO) resulted in high survival rates, with DMSO alone achieving a survival rate of 96 ± 5%, confirming that the solvent itself was non-toxic. CBD-DMSO at concentrations of 25, 50, and 100 μM maintained elevated survival rates (94 ± 7%, 96 ± 6%, and 91 ± 6%, respectively), indicating that CBD dissolved in DMSO did not negatively impact nematode viability.

Similarly, the NE and CBD-NE formulations demonstrated high biocompatibility. The NE formulation alone resulted in a survival rate of 93 ± 7%, confirming its safety in *C. elegans*. CBD-NE at concentrations of 25, 50, and 100 μM yielded survival rates of 98 ± 4%, 96 ± 7%, and 94 ± 11%, respectively, all not statistically different from the control and DMSO-treated groups. These results indicated that the NE formulation did not induce significant toxicity and was effective in delivering CBD without compromising nematode viability.

In contrast, the CS-NE and CBD-CS-NE formulations exhibited pronounced lethality in *C. elegans*. This finding stands in direct opposition to the results from the *in vitro* cytotoxicity assay in HaCaT keratinocytes (Figure 7), where the same formulations showed no significant toxicity. This discrepancy underscores a critical limitation of isolated 2D cell cultures, which lack the integrated physiology of a whole organism, including protective barriers, digestive systems, and neuronal signaling [33]. The severe toxicity in *C. elegans* suggests a mechanism that requires systemic interaction or affects organs not represented in a keratinocyte monolayer.

The results demonstrated that both DMSO-based and NE-based CBD formulations were well-tolerated, with survival rates consistently above 90%, even at the highest concentration tested (100 μM). These findings indicate that CBD did not exert significant toxicity in the *C. elegans* model. In contrast, the CS-NE formulation and its CBD-loaded variants exhibited severe toxicity, resulting in complete or near-complete loss of nematode viability at all tested concentrations. This outcome suggests that chitosan, rather than CBD, was the primary factor contributing to observed toxicity.

The toxicity associated with the chitosan-based formulations may be linked to chitosan’s interaction with *C. elegans* physiology, potentially involving disruption of protective barriers or interfering with key internal processes. We propose two non-mutually exclusive mechanistic hypotheses to explain the specific toxicity of the chitosan-stabilized nanoemulsions: disruption of the nematode cuticle and intestinal barrier, and neurological or systemic toxicity via chitosan uptake.

The positively charged chitosan polymer, freely available in the nanoemulsion’s aqueous phase, could extensively interact with the negatively charged glycoproteins of the nematode cuticle and intestinal lining. This interaction may compromise these critical protective barriers, leading to a loss of osmotic integrity, desiccation, and uncontrolled influx of the formulation components, culminating in rapid organismal death. This mode of action would be absent in cultured cells grown on a plastic substrate [34].

Alternatively, chitosan or chitosan-coated nanodroplets may be ingested and internalized by *C. elegans*, potentially interfering with essential neuronal or metabolic pathways. Cationic polymers like chitosan are known to interact with cellular membranes and can disrupt mitochondrial function or induce oxidative stress in a tissue-specific manner; effects that could be lethal in a whole organism but not immediately apparent in a viability assay on resilient epithelial cells. Previous studies have reported that chitosan-based formulations, including nanoparticles and nanocapsules, can exhibit dose-dependent toxicity, with chitosan coatings contributing to higher mortality compared to other polymeric carriers [33].

A significant limitation of this study must be acknowledged. The chitosan solution was prepared in 1% acetic acid, and the final nanoemulsions had a pH between 4.7–4.9. While the *in vitro* cell medium was strongly buffered, the *C. elegans* exposure medium (M9 buffer) has limited buffering capacity. Therefore, the contribution of residual low pH and free acetic acid to the observed lethality cannot be ruled out and represents a major confounding factor. The acidic environment itself could cause severe physiological stress. Future work must include appropriate pH-matched controls to decouple the effects of chitosan from those of low pH and acetic acid.

Despite this limitation, the data clearly indicate that the chitosan component is the primary driver of toxicity, as neither the NE (without chitosan) nor CBD alone induced adverse effects. The results compellingly demonstrate that while the plain nanoemulsion (NE) is a biocompatible carrier for CBD in this model, the incorporation of chitosan as a stabilizer creates a formulation with unacceptable *in vivo* toxicity under these experimental conditions. This highlights the necessity for stringent *in vivo* safety screening even when *in vitro* results are favorable and underscores that chitosan’s safety profile is profoundly dependent on its formulation, concentration, and the biological context of its application. Future formulations must either employ rigorous purification steps to eliminate acidic residues or utilize alternative, biocompatible stabilizers for intended dermal use.

## 4. Conclusions

This study successfully developed and characterized cannabidiol-loaded argan oil nanoemulsions, both with and without chitosan stabilization, as a potential topical treatment for psoriasis. The formulations exhibited favorable physicochemical properties, with the chitosan-coated system (CBD-CS-NE) showing enhanced viscosity, a positive surface charge, and a sustained, diffusion-controlled drug release profile, all desirable traits for a dermal delivery system. Crucially, *in vitro* assessment confirmed the biocompatibility of these formulations with human keratinocytes (HaCaT cells). However, a critical and contrasting finding emerged from the *in vivo* toxicity assessment in *C. elegans*, where the chitosan-containing formulations induced significant lethality. Therefore, the path forward must prioritize biocompatibility optimization without sacrificing functional performance. In summary, while the plain nanoemulsion (CBD-NE) presents a safe and effective delivery platform, the chitosan-stabilized version (CBD-CS-NE) requires targeted reformulation. This work provides a clear rationale and a specific roadmap for achieving a therapeutically effective and systemically safe topical nanoemulsion system, highlighting the indispensable role of integrated *in vitro*–*in vivo* assessment in nanocarrier development. Overall, the findings establish CBD-loaded argan oil nanoemulsions, particularly the chitosan-stabilized variant, as promising topical delivery platforms. Their favorable physicochemical properties, controlled release, and enhanced skin permeation support their potential for further development as therapeutics for inflammatory skin conditions such as psoriasis, pending validation in disease-specific efficacy models.

## Data Availability

The raw data supporting the conclusions of this article will be made available by the authors on request.

## Abbreviations

The following abbreviations are used in this manuscript:

ANOVA	Analysis of Variance
BOD	Biochemical Oxygen Demand
*C. elegans*	*Caenorhabditis elegans*
CBD	Cannabidiol
CBD-CS-NE	chitosan-stabilized CBD nanoemulsions
CBD-NE	CBD loaded nanoemulsion
CBM	Cannabis-Based Medicine
CS	Chitosan
CS-NE	Chitosan-stabilized nanoemulsion
DDA	Deacetylation Degree
DLS	Dynamic Light Scattering
DMSO	Dimethyl sulfoxide
*E. colis*	*Escherichia coli*
EDTA	Ethylenediaminetetraacetic Acid
ELS	Electrophoretic Light Scattering
EMEM	Eagle’s Minimum Essential Medium
ER	Enhancement Ratio
FCS	Fetal Calf Serum
HLB	Hydrophilic-Lipophilic Balance
HPLC	High-Performance Liquid Chromatography
ISD	Inflammatory Skin Disease
Mw	Molecular weight
NE	Nanoemulsion
NGM	Nematode Growth Medium
PBS	Phosphate-Buffered Saline
PDI	polydispersity index
PIC	Phase Inversion Composition
R^2^	Coefficient of determination
SM	Surfactant Mixture
UHPLC	Ultra High-Performance Liquid Chromatography
ζ-potential	Zeta potential
